# A Suggestion for the American Medical Association

**Published:** 1873-02-15

**Authors:** 


### MEDICAL EXAMINER.

*A Semi-Monthly Journal of Medical Sciences.*

EDITED BY

N. S. DAVIS, M. D., AND F. H. DAVIS, M. D.

Chicago, February 15th, 1873.

### EDITORIAL.

### A SUGGESTION FOR THE AMERICAN MEDICAL ASSOCIATION.

If the leading spirits in the American Medical Association wish an example of a truly *scientific* and *valuable* National Association, and of the kind of professional labor which, alone, will command the respect and enlist the sympathy of medical men throughout the country, we commend to them a perusal of the first pages of the *American Journal of Insanity* for October, containing the proceedings of the Association of Medical Superintendents of American Institutions for the Insane.

But ten pages are devoted to the necessary business of the Association, and a hundred and twenty to very interesting and readable discussions on legitimate scientific topics. The editor of the Chicago MEDICAL EXAMINER, in his examination of the various propositions for improving the organization and working power of the (American Medical) Association will please note.—*Editorial in Boston Medical and Surgical Journal, Dec. 12th*, 1872.

May it please our *confrere* of the Boston *Journal*, we have *noted.* And what is the result? Why are the Procedings of the Association of Medical Superintendents of Institutions for the Insane largely composed of "interesting and valuable discussions," to the exclusion of much other irrelevant matter?

Is it because there is anything peculiar in the plan of organization of that Association, its rules or by-laws? Not at all. It is simply because the meetings of that organization are attended by less than *three-score* of members, all engaged in one special department of professional work, and they secure adequate reports of their discussions.

On the other hand, the meetings of the American Medical Association are attended by from three hundred to six hundred members, representing every department of the healing-art; among whom the representatives of the various *specialties* would, alone, outnumber the membership of the Association of Superintendents of Insane Institutions.

That scientific papers can not be read, patiently listened to, and intelligently discussed, in such an assembly, must be apparent to every thoughtful mind.

And such was amply demonstrated to be the case during the first five or six years of the existence of the American Medical Association. The senior editor of THE MEDICAL EXAMINER made due *note* of the important fact at the time; and instead of sitting down in his editorial sanctum and grumbling about it, and writing down the Association a failure, he made an effort to correct the evil, by dividing the Association into Sections, each of which should represent an important department of medicine, and take charge of the reading and discussion of all papers and topics relating to such department. The general meetings were to be restricted to a few hours each morning, and the rest of the day to work in the several Sections.

He expected that each of these Sections would become permanently organized, with efficient officers, permanent records, and be provided with as suitable rooms to hold their meetings in as the Association itself. Indeed, when the plan first went into operation, at New Haven, in 1860, he anticipated that a very few years would be sufficient to make the practical working of each of the subordinate *Sections* of the Association as complete and efficient as the Association of Medical Superintendents now is. But so imperfectly have these Sections been understood, and so silent has been the medical press of the country concerning them, that scarcely more than half of the local Committees of Arrangements appointed from year to year have known how many Sections there were, or provided any suitable rooms for their accommodation. If the editors of the Boston *Journal*, of the New York *Medical Record*, and of a score of other influential journals in different parts of the country, "will please *note*" these facts, and immediately commence diffusing a correct knowledge of the nature, objects, and importance of the Sections among their readers, and aid us in getting the Committee of Arrangements for the next meeting in St. Louis, not only to provide good rooms for the Sections, and a bulletin board on the platform, at the first general morning session, announcing the papers to be read, or questions to be discussed, in each Section in the afternoon, but also to employ a good reporter for each Section meeting, as suggested in THE EXAMINER two or three months since, they will have, as an aggregate result, not one hundred and twenty, but three hundred pages of "interesting and valuable discussions" for every ten pages devoted to ordinary miscellaneous business. What say you, brethren?

